# One-Year Outcomes After Belatacept Conversion in Adolescent Kidney Transplant Recipients

**DOI:** 10.1016/j.ekir.2025.03.016

**Published:** 2025-03-17

**Authors:** Charlotte Duneton, Roshan George, Rochelle Liverman, Anne-Laure Sellier-Leclerc, Beatrice Beauval, Véronique Baudouin, Elodie Cheyssac, Barry Warshaw, Julien Hogan, Rouba Garro

**Affiliations:** 1 Pediatric Nephrology, Dialysis, and Transplantation Department, Robert Debré University Hospital, APHP, Paris Cité University, Paris, France; 2 Pediatric Nephrology Department, Emory School of Medicine and Children’s Healthcare of Atlanta, Georgia, USA; 3 Pediatric Nephrology Department, Mother-Child University Hospital, Lyon, France; 4 Pediatric Nephrology Department, Félix Guyon University Hospital, La Réunion, France; 5 INSERM U976, Université Paris Cité, Paris, France; 6 Paris Translational Research Center for Organ Transplantation, INSERM, UMR-S970, Université Paris Cité, Paris, France

**Keywords:** belatacept, costimulation blockade, kidney transplantation, outcomes, pediatrics

## Abstract

**Introduction:**

Belatacept (CTLA4-Ig) has shown efficacy in adult kidney transplantation (KT) recipients (improved graft and patient survival and reduced *de novo* donor-specific antibody [DSA]) compared with calcineurin inhibitors (CNIs). Its long-term benefits and monthly i.v. administration have raised interest in pediatric use, particularly in adolescents, who face an increased risk of graft loss because of nonadherence. However, data on belatacept use in pediatrics are limited. Our objective was to report the 1-year outcomes of all adolescents (*N* = 45) who underwent CNIs-to-belatacept conversion between 2018 and 2021, in a multicenter retrospective study conducted in the USA and France.

**Methods:**

Indications included long-term CNI avoidance because of toxicity (e.g., histological changes, posttransplant diabetes, and tremors), suboptimal creatinine, or to improve adherence. One-year outcomes were compared with a propensity-matched cohort of adolescents remaining on CNIs.

**Results:**

The median age was 17 years. Rejection occurred in 11 of 45 patients (24%) at a median of 10 months postconversion (7 T-cell–mediated rejections, 3 antibody-mediated rejections, 1 mixed). Belatacept was discontinued in 3 of 11 patients whereas CNIs were added in 2 of 11. Rejection and *de novo* DSA rates did not differ between patients on belatacept and patients on CNIs. At the individual level, estimated glomerular filtration rate (eGFR) in patients on belatacept without rejection increased significantly (median + 19%), compared with rejectors (−3%, *P* = 0.03) and patients on CNI (nonrejectors: −11%, *P* = 0.0006; or rejectors: −14%, *P* = 0.012). No apparent increase in infectious complications was observed.

**Conclusion:**

Although rejection rates in pediatric patients on belatacept seemed higher than in adult cohorts, they were not significantly different from adolescent patients on CNI, underscoring the challenges related to nonadherence in this population. Tailored immunosuppression strategies, including belatacept, may offer benefits for selected adolescents, but further studies are necessary to define optimal patient selection.

## Graphical abstract

**Figure:**
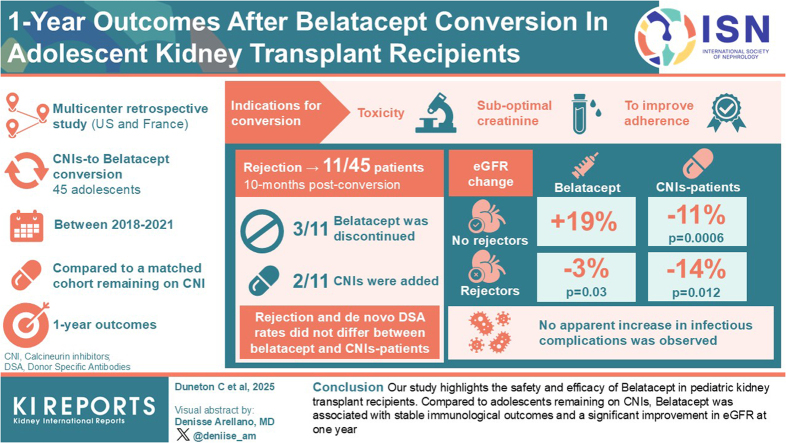

See Commentary on Page 1616

KT is the gold-standard treatment for end-stage renal disease, offering an increase in life expectancy compared with survival on dialysis.^1^ Beyond improved survival, pediatric KT recipients display an improved quality-of-life.^2^, ^3^, ^4^ CNIs have held the central role in immunosuppression since the 1980s.^5^, ^6^, ^7^ Unfortunately, prolonged exposure to CNIs is associated with nephrotoxicity and complications such as diabetes mellitus, dyslipidemia, gingival hyperplasia, alopecia, and hypertension. These adverse effects have significantly contributed to elevated rates of cardiovascular morbidity, increased mortality, and a decline in graft function over time within this patient population.^8^, ^9^, ^10^, ^11^ The quest for alternative regimens to mitigate these side effects has become critical, particularly in pediatric recipients who are expected to remain on immunosuppression for a longer duration compared with adult patients.

Belatacept is a costimulation blocker binding to CD80/86 receptors on antigen-presenting cells to prevent CD28-mediated T-cell activation. Initially, belatacept was used in clinical trials as a *de novo* therapy (administered immediately after transplantation) for adult kidney recipients. In this indication, belatacept has demonstrated its ability to improve graft function in both standard criteria^12^ and extended criteria^13^ donor transplants when compared with cyclosporin. Belatacept was also associated with a lower incidence of *de novo* DSA^12^ as well as a decrease in cardiovascular risk (hypertension, diabetes, and dyslipidemia).^14^ However, clinical trials have raised some concerns regarding higher rates and grades of early acute rejection in belatacept-treated patients. Consequently, several studies have investigated *de novo* belatacept and transient tacrolimus therapy using various combination modalities in an attempt to manage the risk of early rejection.^15^^,^^16^ Building on this, several investigators have explored the possibility of switching to belatacept posttransplantation to avoid prolonged exposure to CNIs or as a rescue therapy for recipients experiencing prolonged delayed graft function^17^ or decreased graft function accompanied by severe histological vascular lesions.^18^ These studies have consistently demonstrated improved renal function following both early^17^^,^^19^ and late switches to belatacept.^18^^,^^20^, ^21^, ^22^, ^23^, ^24^ Late conversion to belatacept has also demonstrated favorable effects on the metabolic profiles of KT recipients.^25^^,^^26^

Despite promising results, regulatory approval for belatacept use in pediatrics is still pending from American and European authorities. Furthermore, limited data are available on its use in older children and adolescents, primarily comprising small, single-center, noncomparative series involving <10 patients.^27^, ^28^, ^29^ Pediatric transplant teams grapple with a unique challenge: an increased risk of graft loss during adolescence, which exists regardless of the recipient's age at the time of renal transplantation.^30^^,^^31^ This heightened risk can be attributed in part to the challenging transition from childhood to adulthood, including potential gaps in care attending to the shift from pediatric to adult management. However, it is primarily driven by noncompliance issues^32^ stemming from behavioral changes during adolescence.^33^ Belatacept, with its monthly i.v. administration, presents an intriguing alternative, particularly suited to address this challenge.

Our objective was to report the 1-year outcomes of all patients (*n* = 45 adolescents) who underwent conversion to belatacept therapy between 2018 and 2021, in a multicenter retrospective study conducted in the USA and France. Conversion indication was based on the need for long-term CNI avoidance either because of toxicity (histology, posttransplant diabetes, tremors), suboptimal creatinine, or to improve adherence. Patients and allograft outcomes under belatacept were compared with a matched cohort of patients who remained on CNI therapy.

## Methods

Forty-five patients aged < 20 years underwent belatacept conversion between May 2018 and December 2021 at 1 pediatric nephrology center in the USA (Children’s Healthcare of Atlanta) and 3 centers in France (Robert Debré, Paris; Lyon; and La Réunion). Before conversion, all patients were confirmed to be Epstein-Barr virus positive (EBV+). The protocol used for conversion to belatacept was the same in all centers and is described in Supplementary Figure S1A and B. Belatacept was administered i.v. in the outpatient clinic with 5 mg/kg every 2 weeks for the first 5 injections (days 1, 15, 29, 43, and 57) and then every 28 days thereafter.^34^ Patients converted to belatacept within the first 3 months posttransplant (early conversion), received a higher first-dose of belatacept (10 mg/kg). CNI doses were reduced in 3 steps of 25% at each belatacept injection as follows: 75% of CNI doses on day 1, 50% on day 14; 25% on day 28, and cessation of CNIs on week 6. Simultaneously, doses of mycophenolate mofetil (MMF) were increased by 50% on day 14 and doubled upon withdrawal of CNIs on week 6.

Histological data (from both surveillance and indication biopsies) were collected before and after the initiation of belatacept. Patients' eGFR (Schwartz *et al.*^35^ formula), urine protein-to-creatinine ratio, viral status (assessed through EBV, cytomegalovirus [CMV], and BK virus PCR, polymerase chain reaction), and DSA were closely monitored monthly. All data were extracted from the medical records.

Patients who underwent belatacept conversion were then compared with a 1:1 matched cohort of patients who remained on CNIs. This control group was chosen from the local databases of 2 centers, Children’s Healthcare of Atlanta in the USA and Robert Debré University Hospital in France, encompassing data from 2012 to 2018. Matching was established using a propensity score, using the following variables: country of residence, age at KT, time since transplantation, donor type, previous history of rejection, and eGFR at the time of inclusion (Supplementary Figure S2). The date of inclusion was defined as the date of first belatacept infusion.

To assess the evolution rate of patients' eGFR, we adopted the following formula: (eGFR at 1 year − eGFR at inclusion) ÷ eGFR at inclusion × 100. All variables were compared using Mann–Whitney and Fisher exact test. Cumulative incidence of rejection and development of *de novo* DSA were compared using the Kaplan-Meier method and log-rank test. Statistical analysis was performed using GraphPad Prism 8, with a significance threshold set at *P* < 0.05. All patients and families were informed about the intended off-label use of belatacept. This study has been approved by the local ethics committee in France and by the institutional review board in the USA. The clinical and research activities being reported are consistent with the Principles of the Declaration of Istanbul as outlined in the “Declaration of Istanbul on Organ Trafficking and Transplant Tourism.”

## Results

### Patients on Belatacept

Between May 2018 and December 2021, 45 patients with a median age of 17.7 (range: 10.3–20.6) years were converted from CNIs to belatacept therapy. Seven of 45 patients underwent early conversion, with a median time of 1 month posttransplant (interquartile range [IQR]: 0.5–1.2 months). Six of 7 patients were prompted by perioperative ischemic injuries, whereas 1 of 7 patients was switched to avoid CNI toxicity (posttransplant diabetes). Patients’ baseline characteristics are summarized in Table 1. Thirty-eight of 45 patients were converted after a median of 4.1 (IQR: 1.7–6.0) years posttransplant. Conversion indication was based on the need for long-term CNI avoidance either because of toxicity (histology, posttransplant diabetes, and tremors: *n* = 13; or suboptimal creatinine: *n* = 12), or to improve adherence (*n* = 13). Patients received mainly first transplants (89%) from deceased donors (67%) and induction treatment with basiliximab (87%) or antithymocyte globulin (13%). All patients were receiving maintenance immunosuppression with MMF, CNIs (tacrolimus, 93%), and steroids, apart from 7 patients who were steroid-free at the time of conversion. CNIs were withdrawn in 4 of /45 patients by a median of 2.4 (IQR: 1.4–6.0) months. In terms of patient history, 31% had experienced rejection episodes previously, with half of these occurring within 4 months before the conversion. Moreover, 38% of patients exhibited preexisting DSA, with a median mean fluorescence intensity of 10,000 (IQR: 1147–31,371). Of the 30 patients who had undergone biopsies within 6 months before conversion, 15 (50%) showed signs of tubulointerstitial and/or microvascular inflammation. Importantly, all patients were seropositive for EBV at the time of conversion, although it is noteworthy that 5 of 45 patients were EBV negative at the time of transplantation. Concerning previous viral complications posttransplant, 2 patients had history of CMV disease, comprising 1 case of CMV pneumonia and 1 gastrointestinal tract infection, both treated with valganciclovir and ganciclovir. In addition, 2 patients had experienced asymptomatic primary CMV infections and had received antiviral treatment, whereas 2 other patients had previously exhibited low titer BK viremia that had resolved before the conversion.

**Table 1:** Baseline characteristics of patients on belatacept (*n* = 45) at conversion

Characteristics	*N* = 45
Male	28
Age at time of conversion (yrs), median (minimum–maximum)	17.7 (10.3–20.6)
Country of residence : USA	27
BMI (kg/m^2^), median (IQR)	22.7 (20.0–26.3)
Deceased donor transplant	30
Prior history of transplant	5
Time from transplant to belatacept start (years), median (IQR)	3.3 (0.9–5.8)
Belatacept indication = nonadherence	13
GFR at initiation of belatacept (ml/min per 1.73 m^2^), median (IQR)	48.7 (32.7–65.3)
Preexisting DSA	17
Median MFI (IQR)	10,000 (1 147–31 371)
Induction therapy (basiliximab)	39
Previous history of rejection	14
Rejection type: ABMR/Mixed/TCMR	5/3/6
Delay prior rejection–belatacept start (months), median (IQR)	3.7 (1.4–28.6)
Inflammation on preconversion biopsy (< 6 months)^a^	15/30
Tubulointerstitial inflammation (*t* + and/or i+) (including TCMR)^a^	6 (1)^a^
Microvascular inflammation (g+ cpt+ C4d+) (including ABMR)^a^	2 (1)^a^
Both (including ABMR)^a^	7 (3)^a^
Chronic vascular lesions and fibrosis on preconversion biopsy^a^	30
Banff cv score (0/1/2/3)	18/9/1/2
Banff ah score (0/1/2/3)	16/4/5/5
IFTA (0/1/2/3)	6/7/7/10
Prior episodes of posttransplant viral complication, *n* (%)	6
(CMV disease/asymptomatic CMViremia/BKviremia)^b^	(2/2/2)
Belatacept dose (mg/kg), median (IQR)	5.3 (5–5.5)
Steroids during conversion to belatacept	38
Everyday/ 1day/2	30/8
AUC MMF per conversion to belatacept, median (IQR)	67.9 (50.0–91.5)
AUC MMF < 50 mg.h/l	9/40
Time to CNI withdrawal (months), median (IQR)	2.4 (1.4–6.0)
Follow-up time (yrs), median (IQR)	1.6 (1.1–2.4)

ABMR, antibody-mediated rejection; AUC, area under the curve; Banff ah score, arteriolar hyalinosis; Banff cv score, vascular fibrous intimal thickening; BKV, BK virus; BMI, body mass index; CMV, cytomegalovirus; CNI, calcineurin inhibitor; DSA, donor-specific antibody; .IFTA, interstitial fibrosis and tubular atrophy; IQR, interquartile range; MFI, mean fluorescence intensity; MMF, mycophenolate mofetil; TCMR, T-cell–mediated rejection.

a The analysis of preconversion biopsies contains only 30 biopsies after exclusion of patients who received an early conversion (*n* = 7) without previous biopsies and biopsies performed more than 6 months before conversion (*n* = 7). Preconversion biopsy could not be performed for 1 patient because of graft hematoma.

b CMV disease consisted of 1 pneumonia and 1 gastrointestinal tract infection.

### Belatacept Outcomes

**Figure 1:**
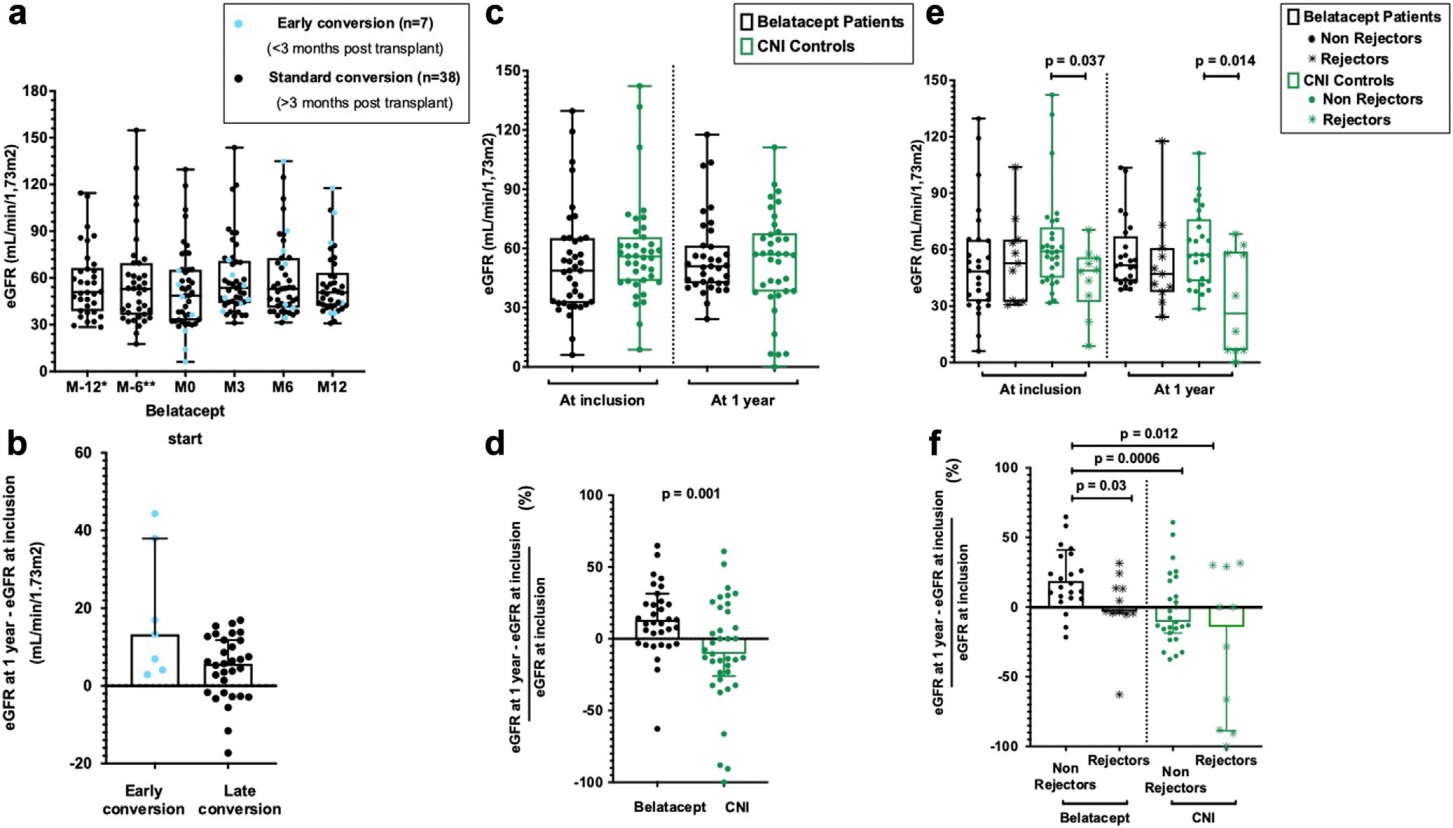
Evolution of eGFR (Schwartz *et al.*^35^ formula). (a) One year before and after belatacept conversion (*n* = 45 patients, including 7 early and 38 late conversions). Data are shown as box and whisker plots with median, interquartile (box) and minimum/maximum values (whiskers). Each point represents a patient. Overall, patients’ graft function remained stable at 1 year after belatacept conversion. ∗*n* = 34 patients at M-12 (exclusion of patients who were converted early, *n* = 7, as well as patients who were transplanted < 1 year before belatacept conversion, *n* = 4). ∗∗*n* = 38 patients at M-6 (exclusion of patients who were converted early, *n* = 7). ∗∗∗*n* = 38 patients at M12 (7 patients with follow-up time < 12 months). (b) Difference between eGFR at 1 year and at baseline (ml/min per 1.73 m^2^) after early (*n* = 7) or late (*n* = 31) conversion to belatacept. Data are shown as median with IQR. Each point represents a patient. At the individual level, patients converted early had a median GFR improvement of 13.3 ml/min per 1.73 m^2^ (IQR: 4.1–37.9), whereas patients converted later had a median GFR improvement of 5.7 ml/min per 1.73 m^2^ (IQR: −1.8 to 11.8). (c) eGFR at inclusion and at 1-year follow-up in patients on belatacept (*n* = 39) versus matched CNI controls (*n* = 39). Data are shown as box and whisker plots with median, interquartile (box) and minimum/maximum values (whiskers). Each point represents a patient. Overall eGFR at 1 year did not differ between patients on belatacept and CNI controls (median: 51 [IQR: 42–61] ml/min per 1.73 m^2^ and 57 [IQR: 38–68] ml/min per 1.73 m^2^, respectively; *P* = 0.88. (d) Evolution rate of eGFR at 1 year in patients on belatacept (*n* = 39) versus matched CNI controls (*n* = 39). Data are shown as median with IQR. Each point represents a patient. When looking at the eGFR evolution rate at the individual level, patients on belatacept showed a significantly higher improvement of eGFR at 1-year (median: +13%, IQR: −3% to 32%) compared with the CNI group (median: −11%, IQR: −26% to 20%, *P* = 0.001). (e) eGFR at inclusion and at 1-year follow-up in rejectors and stable patients in the belatacept (*n* = 39, including 11 rejectors) and CNI control (*n* = 39, including 10 rejectors) groups. Data are shown as box and whisker plots with median, interquartile (box) and minimum/maximum values (whiskers). Each point represents a patient. At 1-year follow-up, there was no significant difference between stable and rejector patients on belatacept. In contrast, patients on CNIs who experienced rejection had significantly lower graft function than stable patients on CNI (a difference that was already present at inclusion for these patients). (f) Evolution rate of eGFR at 1 year in rejectors and stable patients in the belatacept (*n* = 39, including 11 rejectors) and CNI control (*n* = 39, including 10 rejectors) groups. Data are shown as median with IQR. Each point represents a patient. When looking at the eGFR evolution rate at the individual level, stable patients on belatacept showed significant improvement in graft function at 1 year and a significantly higher rate of eGFR progression than patients who experienced rejection on belatacept and patients on CNI (stable or rejectors). CNIs, calcineurin inhibitors; eGFR, estimated glomerular filtration rate; IQR, interquartile range.

Median follow-up time was 1.6 (IQR: 1.1–2.4) years. Overall, eGFR remained stable at 1 year (Figure 1a). On an individual level, patients who underwent early conversion exhibited a median improvement in eGFR of 13.3 (IQR: 4.1–37.9) ml/min per 1.73 m^2^, whereas those who converted later had a median eGFR improvement of 5.7 (IQR 1.8–11.8) ml/min per 1.73 m^2^ at 1 year (Figure 1b). All the patients adhered to the belatacept protocol except for 1 who discontinued treatment because of adherence issues.

Regarding severe viral complications, no cases of posttransplant lymphoproliferative disorder or CMV disease were observed. However, 1 severe BK virus nephropathy was reported, requiring the cessation of belatacept, which subsequently led to the resolution of BK viremia. Belatacept was not resumed because of concerns regarding nonadherence to monthly infusions. In addition, 1 patient presented with symptomatic herpes zoster, which responded well to antiviral treatment. Asymptomatic viral replications were also noted, including EBV (*n* = 5), CMV (*n* = 3), and BK virus (*n* = 2). Among the 5 polymerase chain reaction EBV-positive patients, after increasing to detectable levels, viral load remained stable throughout the balance of the study (∼3 LOG IU/ml). Of the 3 patients with asymptomatic CMV replication, 2 cleared their viral load, including 1 who received valganciclovir treatment, whereas the third patient’s viral load remained stable (∼3 LOG IU/ml). The 2 patients with asymptomatic BK virus replication exhibited spontaneous resolution. Two cases of mild cutaneous fungal infections were observed, resolving after local treatment.

**Figure 2:**
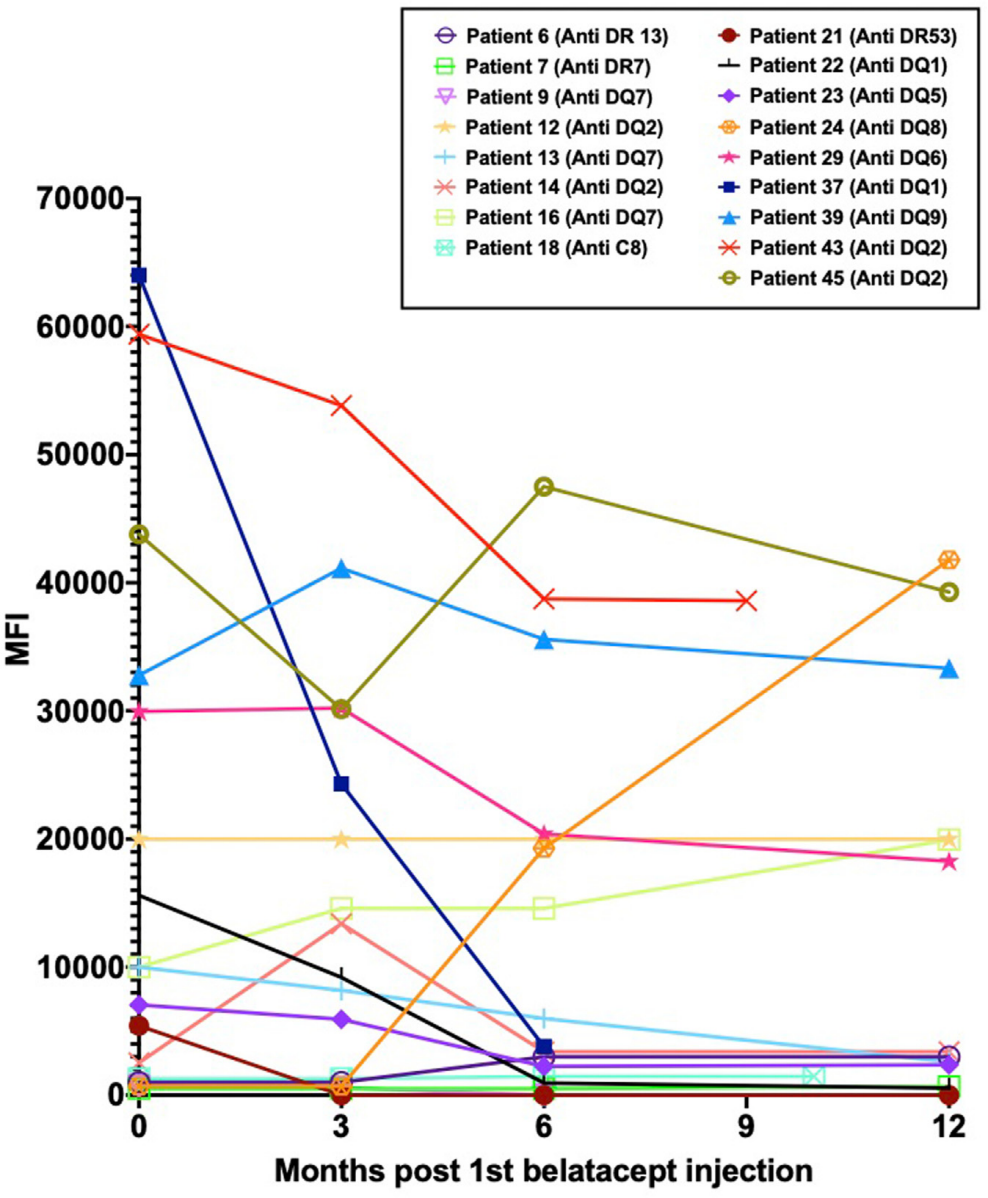
Evolution of preexisting DSA in patients (*n* = 17) at 1-year postconversion to belatacept. Each line represents a patient. If a patient had > 1 DSA, only the immunodominant one is shown in this figure. DSA, donor-specific antibody.

The evolution of DSA in patients who had preexisting antibodies at the time of switch (*n* = 17) is illustrated in Figure 2. Most of them remained stable on belatacept. *De novo* DSA antibodies developed in 3 out of 45 patients (7%), all within the first year after belatacept initiation, while receiving MMF and oral corticosteroids daily (*n* = 2) or every other day (*n* = 1). Of these 3 patients, 2 developed rejection concurrently with the appearance of *de novo* DSA, one of whom experienced mixed T-cell–mediated rejection and ABMR (treated with antithymocyte globulin, i.v. methylprednisolone pulse, and i.v. Ig) and the other ABMR (treated with i.v. methylprednisolone pulse and i.v. Ig). The third patient underwent intensified oral immunosuppressive therapy with MMF and was switched to daily corticosteroids. At the latest follow-up, the first patient's DSA became negative, whereas the other 2 patients showed persistent DSA with decreased mean fluorescence intensity levels (20,000–16,000 and 20,000–10,000, respectively).

Eleven of 45 patients (24%) experienced biopsy-proven acute rejection at a median time of 10.3 months postconversion (7 T-cell–mediated rejections, 3 ABMRs, and 1 mixed). Among these rejections, 4 patients had undergone conversion because of nonadherence, 4 had a previous history of rejection, 4 had preexisting DSA, 6 displayed inflammation in their preconversion biopsies, and 1 was steroid-free at the time of conversion. No significant differences were observed in the baseline characteristics between rejectors and stable patients (Supplementary Figure S2). All 11 patients who experienced rejection received steroid pulses, and additional therapy such as antithymocyte globulin and/or i.v. Ig and/or rituximab was administered as needed, depending on the type of rejection (ABMR, T-cell–mediated rejection, or mixed). All 11 patients showed favorable courses after rejection treatment. After the rejection treatment, the subsequent immunosuppressive therapy varied: as follows:

- In 3 patients, belatacept was discontinued, and the patients returned to the standard immunosuppressive regimen involving tacrolimus, MMF, and steroids.
- In 2 patients, CNIs were added to belatacept treatment.
- In 1 patient, CNIs (which had never been discontinued) were maintained alongside belatacept.

The remaining 5 patients continued with a regimen of belatacept, MMF, and steroids. Detailed characteristics of rejection episodes following belatacept conversion are shown in Table 2.

**Table 2:** Detailed characteristics of rejection episodes following belatacept conversion

Patient	Country	Rejection type	Time since start of belatacept (mos)	Biopsy indication	DSA (on day of biopsy)	Banff	Rejection treatment received	Treatment modification
1	France	Mixed	22	Increased creatinine levels	No	i3 t3 g1 ptc3 C4d1	Steroid pulses × 3 +rituximab × 1 + immunoadsorptions × 10 + monthly i.v. Ig × 6	Still on belatacept
2	France	Cellular	10	Protocol	No	t2 ti3 i-IFTA3	Steroid pulses x 3	Discontinuation of belatacept
3	France	Humoral	10	Increased creatinine levels	No	g1 ptc2 C4d0	Steroid pulses × 3 + rituximab x 1 + immunoadsorptions × 10 + monthly i.v. Ig × 6	Discontinuation of belatacept
4	France	Cellular	6	Protocol	No	t3 ti2 i-IFTA3	Oral corticosteroid therapy	Still on belatacept
5	France	Humoral	19	*De Novo* DSA	Yes	g1 ptc2 C4d0	Steroid pulses × 3 + rituximab × 1 + plasma exchanges × 6 + monthly i.v. Ig × 6	Discontinuation of belatacept
6	USA	Cellular	15	Increased creatinine levels	Yes	i3 t2	Steroid pulses x 3	Still on belatacept
7	USA	Humoral	13	*De Novo* DSA	Yes	g1 ptc1 c4d1	Steroid pulses × 3 + monthly i.v. Ig × 6	Still on belatacept
8	USA	Cellular	5	Protocol	Yes	i3 t3	ATG × 3 + Steroid pulses × 2 + monthly i.v. Ig × 6	Still on belatacept
9	USA	Cellular	13	Increased creatinine levels	Yes	i2 t3	Steroid pulses × 3 + plasmapheresis × 4 + rituximab × 1 + bortezomib × 4 + ATG × 7 + monthly i.v. Ig × 6	Tacrolimus added to belatacept
10	USA	Cellular	7	Protocol	No	i3 t3	Steroid pulses × 3, ATG × 8	Tacrolimus added to belatacept
11	USA	Cellular	4	Protocol	No	i2 t2	Steroid pulses × 3, ATG × 7	Tacrolimus maintained with belatacept

ATG, antithymocyte globulin; DSA, donor-specific antibody.

This table provides an overview of the 11 rejection episodes observed in the belatacept group, including the time of occurrence relative to belatacept conversion, method of detection (e.g., serum creatinine change, protocol or for-cause biopsy), histological classification, and treatment administered.

### Comparison of Patients on Belatacept With Matched CNI Controls

**Figure 3:**
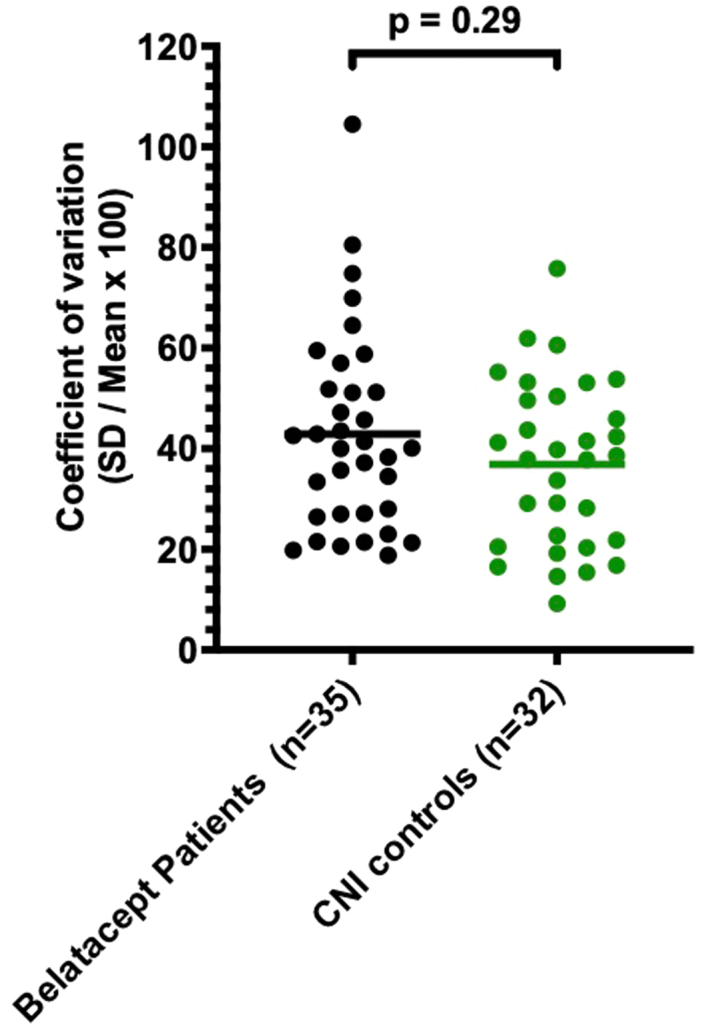
Tacrolimus intrapatient variability over the year preceding conversion to belatacept (or the corresponding inclusion time for CNI controls). Given the challenges of capturing adherence differences directly and incorporating it into the initial matching process, we further analyzed tacrolimus levels intrapatient variability as an indirect marker of adherence. We collected all tacrolimus trough levels (T0) from both the belatacept and CNI control groups during the year preceding conversion (for patients on belatacept) or study inclusion (for patients on CNIs). Of the 39 patients in each group included in the matching, we were able to collect data from 35 patients on belatacept and 32 CNI controls. The mean number of tacrolimus trough levels (T0) collected per patient over the year prior to inclusion was 14 in patients on belatacept and 21 in CNI controls. We calculated intrapatient tacrolimus variability using the coefficient of variation (CV = SD/mean × 100) for each patient as a proxy for adherence, with higher variability in tacrolimus levels suggesting poorer adherence. Importantly, there was no statistically significant difference in the T0 CV between patients on belatacept and those on CNIs, suggesting that adherence patterns were comparable between the 2 groups. CNI, calcineurin inhibitor.

Patients receiving belatacept were compared with a 1:1 matched cohort of CNI-treated patients based on a propensity score, which included factors such as country of residence, age at KT, time since KT, donor type, previous history of rejection, and eGFR at inclusion. The baseline characteristics of patients on belatacept and CNI controls are displayed in Table 3. No significant differences were observed between the groups in terms of matching criteria. However, it is noteworthy that patients on belatacept had a significantly higher prevalence of positive DSA at baseline than CNI controls (36% vs. 10%, *P* = 0.01). Given the challenges of capturing adherence differences directly and incorporating them into the initial matching process, we further analyzed tacrolimus levels intrapatient variability as an indirect marker of adherence. Greater variability in tacrolimus levels has been associated with poorer adherence.^36^ For each patient, we calculated the coefficient of variation (= SD/mean × 100) of tacrolimus trough levels over the year preceding conversion to belatacept (or the corresponding inclusion time for CNI controls). Importantly, no significant difference was observed in coefficient of variation values between the belatacept (median coefficient of variation 40%, IQR: 27%–52%) and CNI groups (median coefficient of variation 38%, IQR: 21%–50%, *P* = 0.29), suggesting comparable adherence patterns in both cohorts (Figure 3).

**Table 3:** Characteristics of matched patients on belatacept (*n* = 39) and CNI controls (*n* = 39) at baseline

*n* (%)	Belatacept	CNI controls	*P*-value
	*n* = 39	*n* = 39	
Country USA	22 (56.4)	24 (61.5)	0.82
Donor type = Deceased	27 (69.2)	33 (84.6)	0.18
Age at transplant (yrs)	14.0 (12.0–16.0)	16.0 (14.0–17.0)	0.07
Time since Tx (yrs)	2.2 (0.8–4.3)	1.8 (0.3–4.0)	0.28
Previous rejection	13 (33.3)	7 (18.0)	0.27
DSA+	14 (35.9)	4 (10.3)	0.01
eGFR (ml/min per 1.73 m^2^), median (IQR)	49 (32–65)	55 (43–66)	0.29
Proteinuria	8 (20.5)	4/26 (15.4)^a^	0.75
Last follow up (mos)	20.4 (13.1–28.3)	18.5 (12.2–26.1)	0.54

CNI, calcineurin inhibitor; DSA, donor-specific antibody; eGFR, estimated glomerular filtration rate.

No significant difference was observed between groups on matching criteria. However, patients on belatacept had significantly more positive DSA at baseline than CNI controls. Mann–Whitney and Fischer’s exact tests were used for comparisons of medians and proportions respectively.

a Missing data on CNI controls proteinuria.

The overall eGFR at 1 year did not exhibit a significant difference between patients on belatacept and CNI controls (median 51 [IQR 42–61] ml/min per 1.73 m^2^ and 57 [IQR 38–68] ml/min per 1.73 m^2^, respectively, *P* = 0.88; Figure 1c). Nevertheless, when examining the eGFR evolution rate on the individual level, patients on belatacept demonstrated a significant improvement in eGFR at 1 year (median: 13%, IQR: −3% to 32%) in comparison to the CNI group (median: −11%, IQR: −26% to 20%, *P* = 0.001; Figure 1d). One graft loss occurred in the CNI group, whereas none occurred in the belatacept group.

**Figure 4:**
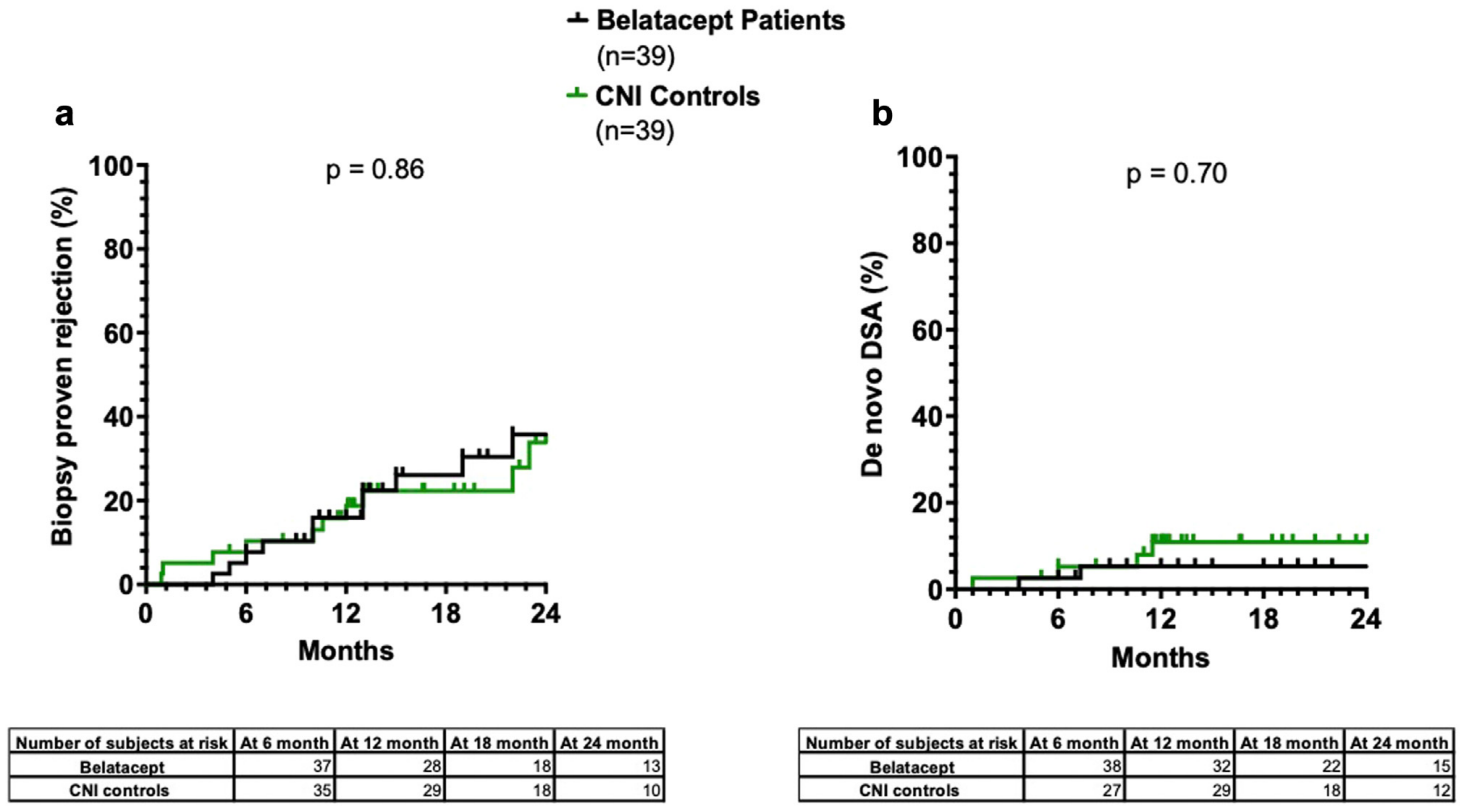
Rejection rate and development of *de novo* DSA in patients on belatacept versus matched CNI controls. Rejection rate and development of *de novo* DSA did not differ between groups. Cumulative incidence of rejection and development of *de novo* DSA were compared using the Kaplan-Meier method and log-rank test. CNI, calcineurin inhibitor; DSA, donor-specific antibody.

Follow-up time was comparable in the 2 groups (1.6 years in the belatacept and 1.7 years in the CNI group, *P* = 0.71). Eleven of 39 patients in the belatacept group experienced rejection episodes compared with 10 of 39 in the CNI group (*P* = 0.86). Two in the belatacept group developed *de novo* DSA, whereas 4 did so in the CNI group (*P* = 0.70). The rates of rejection and *de novo* DSA development did not differ significantly between groups (Figure 4). Overall, at 1-year follow-up, there were no significant differences in eGFR between patients on belatacept who remained free of rejection (52 [IQR: 43–67] ml/min per 1.73 m^2^) and those with rejection (47 [IQR: 38–61] ml/min per 1.73 m^2^, *P* = 0.27). In contrast, Patients on CNI who experienced rejection had significantly lower graft function (26 [IQR 7–59] ml/min per 1.73 m^2^,) than patients on CNI without rejection (57 [IQR: 43–76] ml/min per 1.73 m^2^, *P* = 0.14). This difference was already present at the time of inclusion (Figure 1e). When analyzing the eGFR evolution rate on an individual basis, patients who remained free-of-rejection on belatacept displayed a significant improvement in graft function at 1 year (Figure 1f). In these patients, the median increase in eGFR at 1 year was 19% (IQR: 7%–41%), which was significantly higher than patients who experienced rejection while on belatacept (−3%, [IQR: −4% to 13%, *P* = 0.03) and patients on CNIs (either without rejection: −11%, IQR: −19% to 19%, *P* = 0.0006; or with rejection: −14%, IQR: −89% to 29%, *P* = 0.012).

## Discussion

We present the 1-year outcomes for the largest multicenter cohort of pediatric patients undergoing belatacept treatment to-date. Our findings confirm its safety, showing no apparent increase in infectious complications and no association with a higher risk of rejection when compared with a matched cohort receiving CNIs. At the individual level, patients receiving belatacept displayed a significantly improved eGFR evolution rate at 1 year compared with those remaining on CNIs. The matching was designed to ensure that adolescent patients were compared with their peers, accounting for the similar challenges of nonadherence and increased risk of rejection, unfortunately common at this age. In the absence of randomized controlled trials in the pediatric population, our study offers valuable data regarding belatacept conversion outcomes in adolescent patients.

In response to early reports of an elevated rejection risk associated with costimulation blockade, particularly given the high-risk profile of adolescents, we performed kidney biopsies in most of our patients according to each center protocol (surveillance or indication) allowing us to assess the risk of rejection in this population.

In our cohort, 11 of 45 patients (24%) developed biopsy-proven acute rejection, which is higher than the rates reported in adult cohorts converted to belatacept (4%–8%).^18^^,^^24^ However, this rate was comparable to the rates reported in pediatric cohorts treated with CNI. Varnell *et al.*^37^ reported a rejection rate of 22% after a 2-year observation period in such a cohort of 98 patients (mean age: 13 years, with 27% having a history of rejection and 15% having preexisting DSA). Our study's matched analysis revealed no significant increase in the risk of rejection in patients treated with belatacept compared with those remaining on CNI therapy. Importantly, all patients who experienced rejection achieved resolution with treatment, and we did not observe any graft loss in the belatacept group.

Patients converted to belatacept-based therapy demonstrated a substantial improvement in eGFR rate (+ 13%) at 1 year, which was significantly higher than in matched CNI controls, regardless of their rejection status. This improvement was even more pronounced in patients on belatacept who remained free of rejection. Several adult studies aimed at predicting patients who will benefit from belatacept therapy and who are at higher risk of rejection.^38^, ^39^, ^40^, ^41^ We found no clinical factors predictive of rejection on belatacept in our study. This currently constitutes a limitation for a broader use of belatacept in this population and advocates for the development of new biomarkers to better immunologic risk-stratify our patients. Regarding the development of *de novo* DSA, incidence rates did not differ between our belatacept-converted patients and matched CNI controls, which contrasts with the findings in adult studies.^18^^,^^24^ However, the relatively small size of our cohort and the short follow-up may have limited our ability to detect a difference.

All patients adhered to the belatacept protocol except for 1 who discontinued treatment. The monthly i.v. administration of belatacept offers new possibilities for improving therapeutic compliance in this high-risk population. Although most infusions take place at the hospital, some centers in France have recently begun offering home-based infusions by nurses, particularly in light of the COVID-19 pandemic. This approach could prove beneficial in reducing absenteeism in these patients for whom medical care often poses challenges to schooling.

Another concern with the use of costimulation blockade is an increased risk of EBV replication and posttransplant lymphoproliferative disorder development.^42^ This has led to the contraindication of belatacept in EBV seronegative patients. Given that children are at an increased risk of viral infections and almost half of pediatric recipients are EBV-seronegative at transplant,^35^ this could potentially limit the number of eligible pediatric patients. We did not observe any cases of lymphoproliferation in our cohort, including in the 5 out of 45 patients who were EBV-seropositive at the time of conversion but had been EBV-negative at transplantation.

Several studies have raised concerns about an increased incidence of infections after belatacept conversion,^43^ especially CMV infections in patients with impaired graft function at the time of conversion.^44^^,^^45^ However, our study, though limited in patient numbers, provides reassuring results regarding infections and no symptomatic CMV replication. Patients in our cohort did not systematically receive prophylaxis against *Pneumocystis jirovecii* after switching to belatacept.

It is important to note that most patients in our cohort received basiliximab induction, which may have influenced both rejection and infection rates. Whereas some centers favor depleting agents such as antithymocyte globulin or alemtuzumab (Campath), the use of basiliximab in our population likely contributed to the overall low rate of infectious complications observed. In addition, differences in induction strategies could impact rejection incidence, particularly in the early posttransplant period, and should be considered when comparing outcomes across different centers.

The strength of our study lies in its multicenter and international design, allowing us to report on the largest belatacept-treated pediatric KT cohort to-date and to compare outcomes with a matched cohort of CNI-controls. However, our study has certain limitations, including its retrospective design and a relatively short follow-up time. In addition, other potential beneficial effects of belatacept, such as metabolic and cardiovascular outcomes, were not assessed in our study, or did our study investigate patients' perceived quality-of-life.

In conclusion, our study provides valuable data on the efficacy and safety of belatacept use in adolescents with KT. Although, the overall rejection rate among belatacept-treated children appears higher than in adult cohorts, rejection rates were comparable to adolescents on CNIs. Moreover, consistent with adult data, some patients demonstrated marked improvements in renal function within the first year postconversion. While awaiting the result of an ongoing randomized controlled trial (NCT04877288) evaluating CNI-to-belatacept conversion in adolescents with stable allograft function, our study provides valuable real-life data on the use of belatacept and reassuring safety data regarding the risk of viral infection. Further independent, investigator-initiated studies are essential to determine optimal patient selection and timing for conversion to belatacept, to maximize its potential benefits.

## Disclosure

All the authors declared no competing interests.

## Data Availability Statement

The data that support the findings of this study are available on request from the corresponding author, CD.

## Author Contributions

CD contributed to conceptualization, methodology, data collection, statistical analysis, visualization, and writing (original draft preparation). RG, RL, A-LS-L, BB, VB, and EC contributed to conceptualization and data collection. BW contributed to writing (review and editing). JH contributed to conceptualization, methodology, data collection, statistical analysis, supervision, and writing (review and editing); RG contributed to conceptualization, methodology, data collection, supervision, and writing (review and editing). All the authors discussed the results and contributed to the final manuscript.

## Supplementary Material

Supplementary File (PDF) **Figure S1.** Belatacept conversion protocol. **Figure S2.** Baseline characteristics of rejectors (*n* = 11) and stable patients on belatacept (*n* = 34) at conversion. **Figure S3.** Flow chart.

## Footnotes

Supplementary File (PDF)
**Figure S1.** Belatacept conversion protocol. **Figure S2.** Baseline characteristics of rejectors (*n* = 11) and stable patients on belatacept (*n* = 34) at conversion. **Figure S3.** Flow chart.
