# Supplementary material for: One-Year Outcomes After Belatacept Conversion in Adolescent Kidney Transplant Recipients
Source: Kidney Int Rep. 2025 Mar 17;10(6):1795–805. doi: 10.1016/j.ekir.2025.03.016 (PMC12232964; doi:10.1016/j.ekir.2025.03.016)
Supplement: Supplementary File (PDF) — Figure S1. Belatacept conversion protocol. Figure S2. Baseline characteristics of rejectors (n = 11) and stable patients on belatacept (n = 34) at conversion. Figure S3. Flow chart. [file mmc1.pdf]

SUPPLEMENTARY MATERIAL

Supplementary Figure S1. **Belatacept conversion protocol** (page 2)

Supplementary Figure S2. **Baseline characteristics of rejectors (n=11) and stable patients on belatacept (n=34) at conversion** (page 4)

Supplementary Figure S3. **Flow chart** (page 4)

A

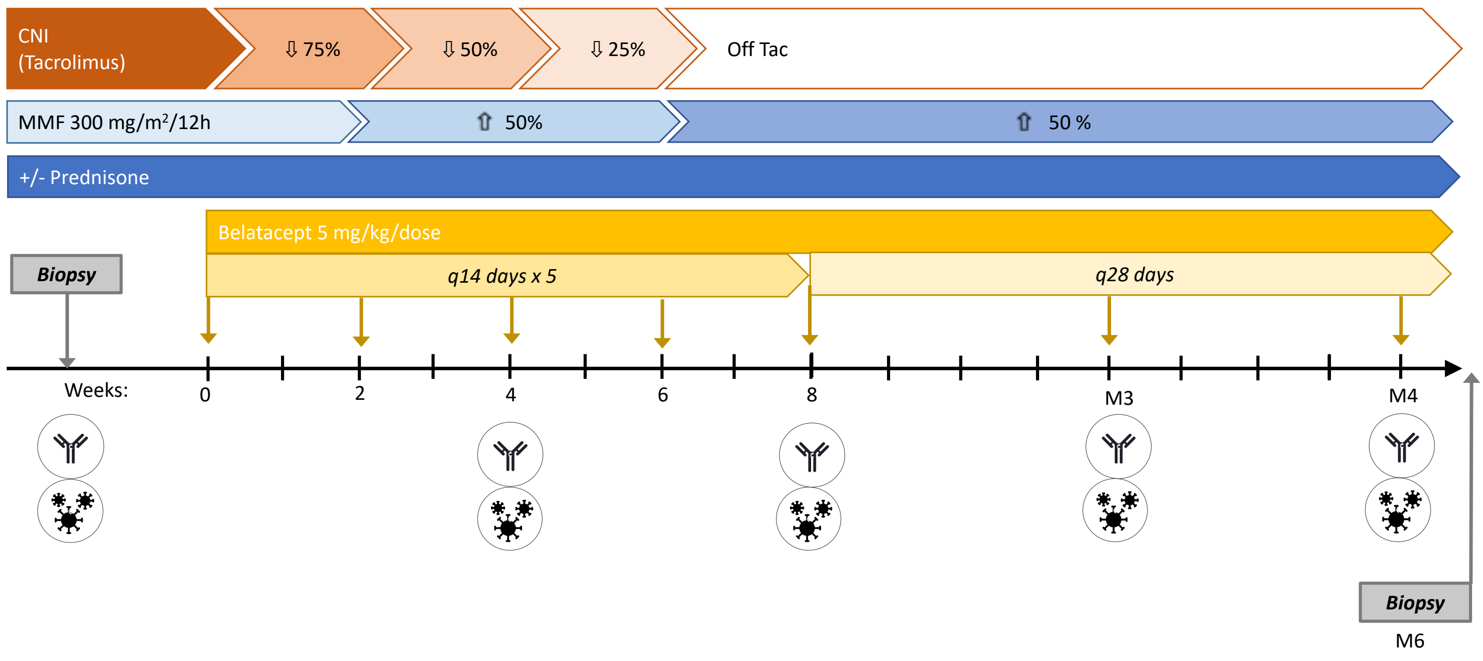
CNI
(Tacrolimus)
⇩ 75%
⇩ 50%
⇩ 25%
Off Tac
MMF 300 mg/m²/12h
⇧ 50%
⇧ 50 %
+/- Prednisone
Belatacept 5 mg/kg/dose
q14 days x 5
q28 days
Biopsy
Weeks:
0
2
4
6
8
M3
M4
Biopsy
M6

B

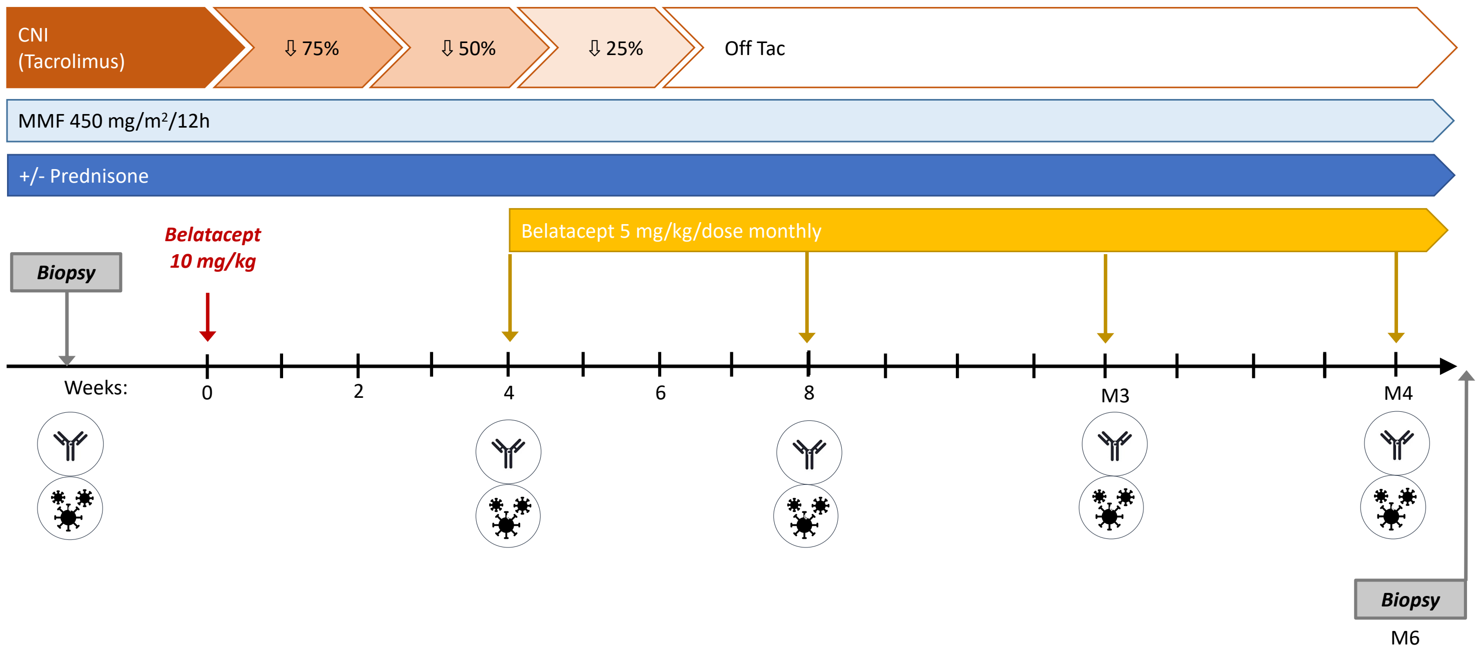
CNI
(Tacrolimus)
⇩ 75%
⇩ 50%
⇩ 25%
Off Tac
MMF 450 mg/m²/12h
+/- Prednisone
Belatacept
10 mg/kg
Belatacept 5 mg/kg/dose monthly
Biopsy
Weeks:
0
2
4
6
8
M3
M4
Biopsy
M6

Supplementary Figure S1. Belatacept conversion protocol

A. The first 5 belatacept injections were administered at 5mg/kg/dose (10mg/kg/dose if early conversion, < 3 months post-transplant) every 2 weeks, then monthly. CNI doses were progressively decreased by 25% at each belatacept injection and stopped at injection n°4. MMF doses were increased by 50% at injection n°2 and doubled at CNI withdrawal. Allograft biopsies were performed before and 6 months after belatacept initiation. Patients' viral status (PCR EBV, CMV, BKv) and DSA were monitored monthly.

B. Patients who were converted to Belatacept withing the first three months post transplant (early conversion), received a higher first dose of belatacept (10 mg/kg) followed by monthly dose of 5 mg/kg.

	Rejectors (N = 11)	Stable (N = 34)	p value
Male	9	19	0.16
Age at time of Bela conversion (years), median (min-max)	17.7 (15.8 – 19.2)	17.6 (16.2 – 19.1)	0.83
Country of residence : USA	6	21	0.73
BMI (kg/m2), median (IQR)	22.5 (19.9 – 26.3)	23.4 (21.5 – 26.8)	0.54
Deceased donor transplant	7	23	>0.99
Prior history of transplant	2	3	0.58
Time from transplant to belatacept start (years), median (IQR)	1.8 (1.1 – 4.5)	3.3 (0.8 – 6.0)	0.73
Belatacept indication = nonadherence	4	9	0.70
GFR at initiation of belatacept (ml/min/1.73m^2), median (IQR)	52.7 (32.3 – 65.2)	48.4 (33.1 – 65.3)	0.76
**Pre-existing DSA**	4	13	>0.99
Median MFI (IQR)	17 800 (4 275 – 29 591)	7 065 (1147 – 36 872)	0.85
**Induction therapy** (basiliximab)	9	30	0.62
**Prior history of rejection**	4	10	0.72
Type : ABMR / Mixed / TCMR	2 / 0 / 2	3 / 3 / 4	
Delay prior rejection – belatacept start (months), median (IQR)	2.8 (1.6 – 3.9)	9.4 (1.3 – 35.1)	0.45
**Inflammation on pre-conversion biopsy (<6 months, n=30)***	6 / 10	9 / 20	0.70
Tubulo-interstitial inflammation (t+ and/or i+) (*including **TCMR**)	2	4 **(*1)**	
Microvascular inflammation (g+ cpt+ C4d+) (*including **ABMR**)	1	1 **(*1)**	
Both (*including **ABMR**)	3 **(*1)**	4 **(*2)**	
**Chronic vascular lesions and fibrosis on pre-conversion biopsy (n=30)***			
cv score (0 / 1 / 2 / 3)	5 / 5 / 0 / 0	13 / 4 / 1 / 2	0.46
ah score (0 / 1 / 2 / 3)	5 / 1 / 1 / 3	11 / 3 / 4 / 2	>0.99
IFTA (0 / 1 / 2 / 3)	3 / 3 / 1 / 3	3 / 4 / 6 / 7	0.37
**Prior episodes of post-transplant viral complication**	3	3	0.15
(CMV disease** / asymptomatic CMViremia / BKviremia)	(1 / 1 / 1)	(1 / 1 / 1)	
Bela dose (mg/kg), median (IQR)	5.2 (5.1 – 5.4)	5.3 (4.9 – 5.6)	0.57
Steroids during conversion to belatacept	10	28	0.67
Everyday / 1 day / 2	10 / 0	20 / 8	0.08
AUC per conversion to belatacept, median (IQR)	64.9 (55.5 – 78.5)	77.5 (49.8 – 111.3)	0.52
AUC < 50 mg.h/l	2 / 10	7 / 30	0.70
Time to CNI withdrawal (months), median (IQR)	2.5 (1.4 – 3.9)	2.4 (1.4 – 6.5)	0.70
Follow up time (years), median (IQR)	1.6 (1.2 – 2.0)	1.7 (1.1 – 2.4)	0.71

Supplementary Figure S2. Baseline characteristics of rejectors (n=11) and stable patients on belatacept (n=34) at conversion

No significant difference was found between rejectors and stable patients' characteristics at the time of conversion to belatacept. Mann Whitney and Fischer's exact tests were used for comparisons of medians and proportions respectively.

*The analysis of pre-conversion biopsies contains only 30 biopsies after exclusion of patients who received an early conversion (n=7) and biopsies performed more than 6 months before conversion (n=8). ** CMV disease consisted of 1 pneumonia and 1 gastro-intestinal tract infection. BMI (Body Mass Index). QD (every day). QOD (every other day).

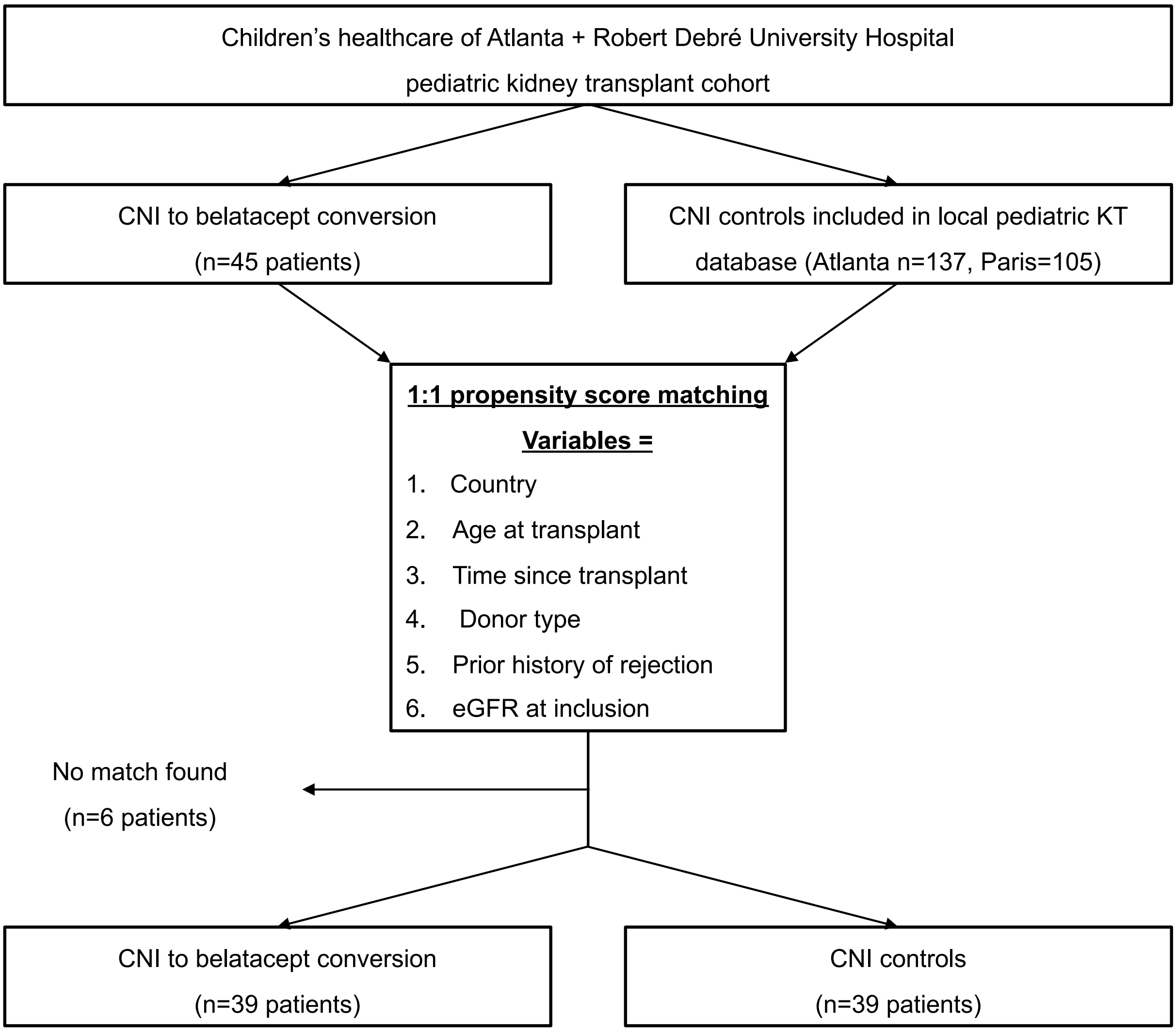

Supplementary Figure S3. Flow chart

Patients on Belatacept were compared to a 1:1 matched cohort of patients remaining on CNI and included in two center's local database from 2012 to 2018 (Children's healthcare of Atlanta in the US and Robert Debré University Hospital in France) based on a propensity score. Variables included: country of residence, age at KT, time since KT, donor type, prior history of rejection and eGFR at inclusion. The date of inclusion was defined as the date of first belatacept injection. CNI controls' biological results and clinical characteristics were matched to those of the belatacept patients on that day.
